# Nitrogen Emission and Deposition: The European Perspective

**DOI:** 10.1100/tsw.2001.285

**Published:** 2001-12-12

**Authors:** Jan Willem Erisman, Peringe Grennfelt, Mark Sutton

**Affiliations:** ^1^ ECN, P.O. Box 1, 1755 ZG Petten, The Netherlands; ^2^ IVL, P.O. Box 47 086, S-402 58 Gothenburg, Sweden; ^3^ CEH, Bush Estate, Penicuik, Midlothian, EH26 0QB, Scotland, UK

**Keywords:** ammonia, nitrogen oxides, N_2_O, emission, deposition, Europe, critical loads, exceedances, policy, scenarios

## Abstract

Europe has been successful in reducing the emissions of several nitrogenous pollutants over recent decades. This is reflected in concentrations and deposition rates that have decreased for several components. Emissions of nitrogen containing gases are estimated to have decreased in Europe by 10%, 21%, and 14% for NO, NOx, and NH3, respectively, between 1990 and 1998. The main reductions are the result of a decrease in industrial and agricultural activities in the east of Europe as a result of the economic situation, measures in the transport sector, industry and agricultural sector, with only a small part of the reduction due to specific measures designed to reduce emissions. The reduction is significant, but far from the end goal for large areas in Europe in relation to different environmental problems. The Gothenburg Protocol will lead to reductions of 50 and 12% in 2010 relative to 1990 for NOx and NH3, respectively. The N2O emissions are expected to grow between 1998 and 2010 by 9%. Further reductions are necessary to reach critical limits for ecosystem protection, air quality standards and climate change. Emissions of nitrogen compounds result from an overload of reactive nitrogen, which is produced by combustion processes, by synthesis of ammonia or by import from other areas as concentrated animal feeds. Although some improvements can be made by improving the efficiency of combustion processes and agricultural systems, measures to reduce emissions substantially need to be focused on decreasing the production or import of reactive N. Reactive N ceilings for regions based on critical limits for all N-related effects can help to focus such measures. An integrated approach might have advantages over the pollutant specific approach to combat nitrogen pollution. This could provide the future direction for European policy to reduce the impacts of excess nitrogen.

## INTRODUCTION

The global nitrogen cycle is being perturbed in many ways. Fossil fuel combustion, mineral fertilisers and livestock manures all provide major sources of fixed reactive nitrogen (N). This leads to a cascade of effects as the N is transported and transformed through the environment (e.g., [1,2,3]). Emissions to the atmosphere result in impacts on human health, visibility, crop damage, regional acidification and eutrophication, as well as global warming, while releases to land result in eutrophication of both fresh and coastal waters. Measures have been taken to decrease effects of N related to air, soil and (ground) water. Most of the measures in Europe have so far focused on decreasing human and plant exposure to N pollutants and to decrease ecosystem loads leading to acidification and eutrophication. Countries have agreed on reductions by signing different protocols developed under the Convention on Long Range Transboundary Air Pollution (CLRTAP)[4]. Table 1 gives an overview of different targets. The last protocol, the Gothenburg Protocol, is unique in the sense that it establishes reductions of four pollutants to abate three effects (acidification, eutrophication and the effects of tropospheric ozone on human health and vegetation). The protocol, which at present has been signed by 29 European countries together with the U.S. and Canada, is based on a gap-closure method aiming to decrease the spatial exceedance of critical loads and levels in the most cost-efficient way. The most cost effective way to bridge gap between deposition and critical loads is found by integrated assessment modeling approach by IIASA in Austria. The countries themselves estimate the critical loads. The agreed reductions for the EU member states are listed in Table 1. The outcome of the Gothenburg protocol is an expected reduction in the European (except Russia) emissions of nitrogen oxides of approximately 50% for the period 1990–2010. The corresponding figure for ammonia is 12%. For the U.S. and Canada there are no defined emission reduction targets under this protocol.

Apart from the protocols, the EU member states have to fulfill several Directives. In May 1999 the European Commission presented a proposal for a Directive on national emission ceilings (NECD) for the same pollutants as CLRTAP and, for the first time, for ammonia[5]. The proposed Directive uses a similar approach as the Second Sulphur Protocol, but extends it to include reduction in exceedance of critical limit values for ozone for human health and ecosystems. The approach is the same as used for the Gothenburg protocol. The targets in the NECD proposal (Table 1), which still need to be adopted, are much stricter than currently agreed targets in the EU according to the existing agreements. For many countries, the targets within the NECD are expected to be lower than those agreed upon in the UNECE-CLRTAP protocol. This paper gives an overview of the current emissions of nitrogen, the effect of the policy to reduce emissions and the benefits. Furthermore, options to reach these and possible future targets are discussed.

## EMISSIONS

The range of important nitrogen forms released to the environment from anthropogenic activity is broad but clearly defined, and can be summarised as:

- emissions of nitrogen oxides ($NO_x$) to the atmosphere;
- emissions of ammonia ($NH_3$) to the atmosphere;
- emissions of radiatively active nitrogen, as nitrous oxide ($N_2O$) to the atmosphere;
- production of N containing aerosol, acids and PAN (peroxy-acetyl-nitrate) in the atmosphere;
- emission of oxidised and reduced nitrogen to the soil and groundwater;
- losses of nitrate and other N forms from soils into the freshwater and marine environments.

**TABLE1**
**Air Emission Reduction Targets for the EU[6]**

| Policy/Pollutant | Base year | Target year | Reduction (%) |
|---|---|---|---|
| **UNECE-CLRTAP** | | | |
| Sulphur dioxide[1] | 1980 | 2000 | 62 |
| Sulphur dioxide[4] | 1990 | 2010 | 75 |
| Nitrogen oxides[2] | 1987 | 1994 | Stabilisation |
| Nitrogen oxides[4] | 1990 | 2010 | 50 |
| Non-methane volatile organic compounds (VOC's)[3] | 1987 | 1999 | 30 |
| Non-methane volatile organic compounds (VOC's)[4] | 1990 | 2010 | 58 |
| Ammonia[4] | 1990 | 2010 | 12 |
| **5th Environmental Action Plan** | | | |
| Sulphur dioxide | 1985 | 2000 | 35 |
| Nitrogen dioxide | 1990 | 2000 | 30 |
| Non-methane volatile organic compounds (VOC's) | 1990 | 1999 | 30 |
| **Dir. on Nat. Emission ceilings (NECD), proposed targets[5]** | | | |
| Sulphur dioxide | 1990 | 2010 | 78 |
| Nitrogen dioxide | 1990 | 2010 | 55 |
| Non-methane volatile organic compounds (VOC's) | 1990 | 2010 | 62 |
| Ammonia | 1990 | 2010 | 21 |

[1]Target from the 1994 Second Sulphur Protocol. The different emission ceilings for each Member State correspond to an overall 62 % emission reduction for the EU.
[2]Targets from first $NO_x$ Protocol. These are the same for individual Member States and for the EU.
[3]Targets from NMVOCs Protocol. These are the same for individual Member States and for the EU.
[4]Targets from the multi-pollutant "Gothenburg" Protocol (1 December 1999). The emission reduction target for the EU that correspond to the different emission ceilings for each Member State (as the EU was not formally a signatory to this protocol).
[5]Targets from the European Commission's 1999 proposal for a National Emission Ceilings Directive (NECD). The emission reduction target for the EU that corresponds with different emission ceilings for each Member State is shown.

The last two are partly related to long-range transport of oxidised and reduced nitrogen compounds, or, in agricultural areas to application of fertilisers or manure. The atmospheric emissions of nitrogen oxides (NO and $NO_2$) are from combustion processes where fuel N and molecular N form the combustion air are oxidised into NO, and subsequently reacts with ozone to produce $NO_2$. Major source categories are therefore, transport, industry and energy production. Other sources of NO include soils, particularly where N inputs are high. Ammonia is naturally emitted to the atmosphere from oceans, manure from wild animals and ageing or rotting plants. However, most of the emission on a European scale is of anthropogenic origin. The largest source of $NH_3$ is emission from animal manure followed by fertilizer use[7,8], whereas other sources include vehicles equipped with a three-way catalyst to reduce $NO_x$, fertiliser production, coal combustion, landfill, households and humans (e.g., [9,10]). Nitrous oxide emissions result from fertiliser application, three-way catalyst and industrial activities, such as nitric acid production[6]. Annual emissions reported by parties to the CLRTRAP are summarised by EMEP (Co-operative programme for monitoring and evaluation of the long range transmission of air pollutants in Europe) (e.g., [11]). The European Environment Agency (EEA) interprets emission data, co-ordinates the development of the spatial distribution of emissions and provides information on policies and scenarios. The information provided in this paper is mainly based on these two sources.

The estimated spatial distribution of $NH_3$ and $NO_x$ in 1998 is displayed in Fig. 1. The maps show that emissions are not equally distributed over the surface area: $NO_x$ emissions are largest in cities and industrialised areas, while $NH_3$ emissions are highest in intensive livestock areas. In the cities $NO_2$ and $O_3$ are important pollutants affecting human health. In and close to the intensive livestock areas the environmental effects are most problematic, especially where sensitive receptors are located directly in the source regions. By contrast, some of the most sensitive areas are located far from source areas, and for these the deposition from long-range transboundary

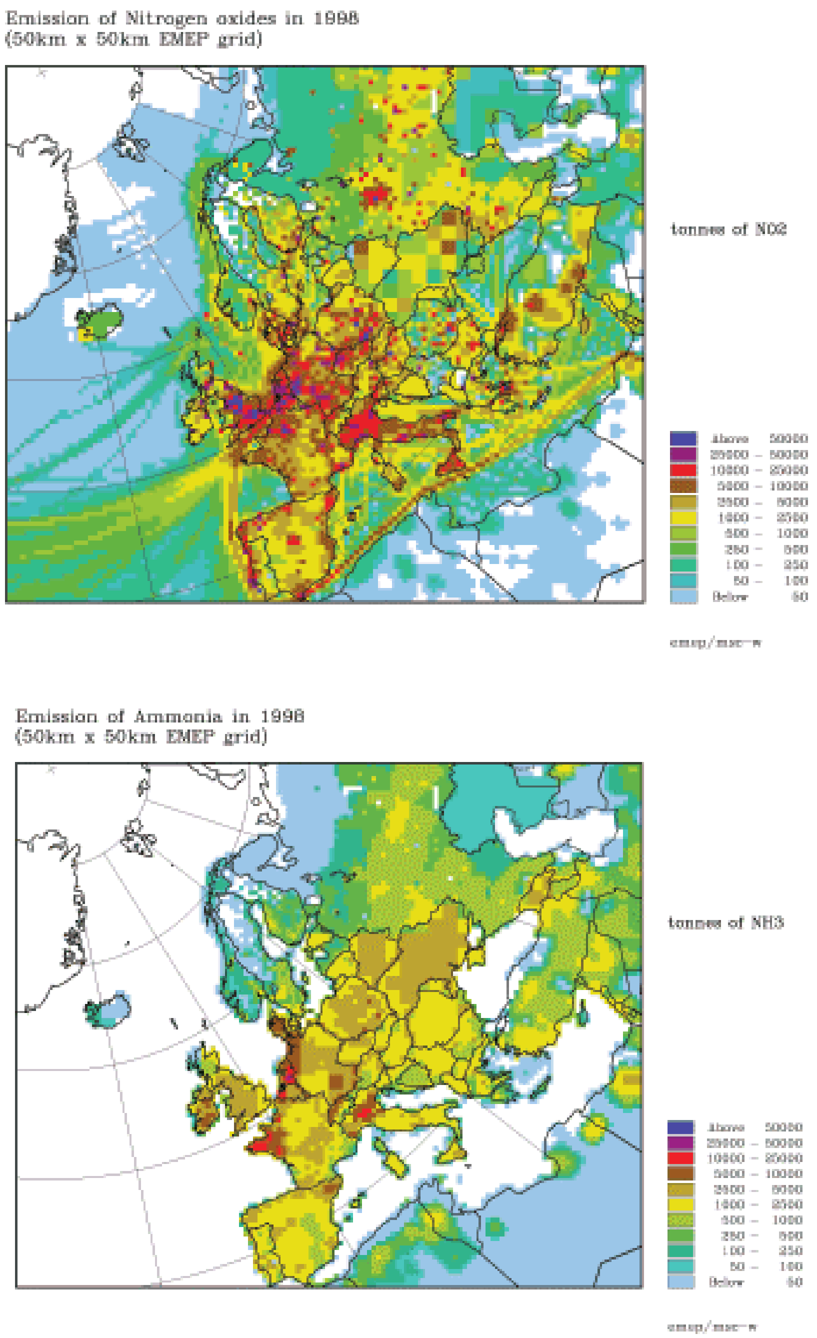

**FIGURE 1.** Estimated emissions of nitrogen oxides (tonnes $NO_2$) (*top*) and ammonia (tonnes $NH_3$) (*bottom*) in 1998 at 50 km resolution in Europe[11].

pollutant transport is critical. If the emissions were distributed equally over the whole of Europe, the critical limits for different effects would not be exceeded anywhere[12]. This, however, does not hold for $N_2O$, because it contributes to accumulated concentration in the atmosphere, which has a long lifetime. Therefore, if the total emission rate exceeds the atmospheric destruction rate, $N_2O$ concentrations will continue to rise and climate change consequences will be amplified.

The protocols (Table 1) have had a major effect on the emission trends in Europe, especially for $SO_2$. European emissions reductions are being made with the clear objective that environmen-

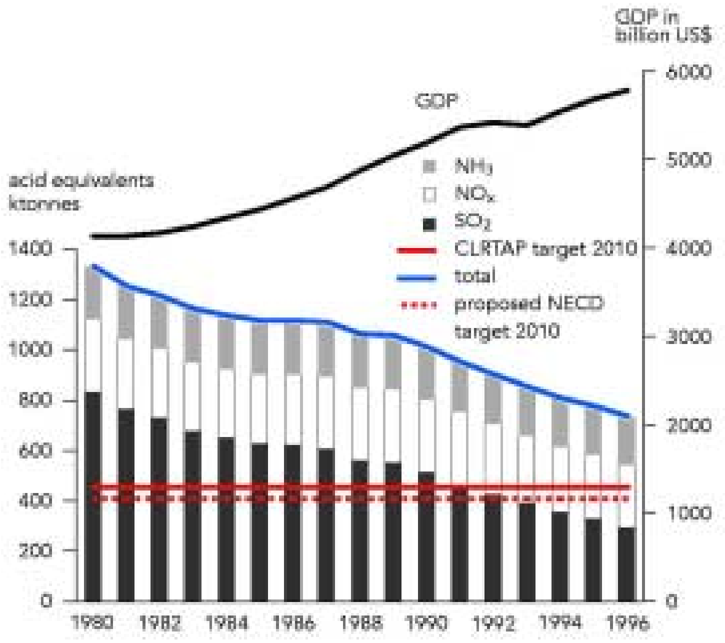

**FIGURE 2.** Trend in European emissions (Mton S or N). Source: EPA[6].

tal loads, exposures and effects should be decreased – the so called "effects-based approach", which was initiated under the Second Sulphur Protocol[13]. Fig. 2 shows the development of total European yearly emissions of acidifying pollutants and the GDP between 1980 and 1996 as reported by EEA[12]. For the purpose of this assessment it is assumed that each molecule of $SO_2$ emission leads to the release of two protons, while $NO_x$ and $NH_3$ emission each lead to one proton being released. The downward trend in acidifying emissions is obvious from this figure, but mainly determined by the decrease in sulphur emissions. The figure also shows that there is a decoupling between economic development, in terms of GDP, and emissions: emissions decrease while GDP increases. This shows that regulations for environmental protection do not have to limit economic growth.

Fig. 3 shows the changes in emissions between 1990 and 1998 for $NO_x$, $NH_3$, and $N_2O$ for those countries under the UNECE Convention that reported their emissions in the two years. The European sulphur emission was reduced by 41% between 1980 and 1998. In the same years $NO_x$ emission was brought down by 21%, mainly after 1990. Ammonia emissions yield very high uncertainties. Until recently only a few countries reported their emissions but the situation has improved and now there seems to be a consistent dataset available in which emission data are available from 1980 and onwards. For $NH_3$ it shows that the emissions went down by 14% between 1980 and 1998. With most countries using standard emission factors for $NH_3$, these differences are largely a result of estimated changes in animal numbers and fertilizer use. Hence the largest reported reductions occurred in eastern Europe following the political changes of 1989–1990, which led to major reductions in agricultural activity (e.g., [14,15]). A few countries, such as Denmark, the Netherlands and Germany have taken measures to reduce $NH_3$ emissions. These include injection of slurry into the soil, coverage of manure storage facilities, development of low-emission housing systems and decrease of N in concentrates. However, for $NH_3$, uncertainties in emissions are very large and even at the country scale current studies have been unable to obtain a satisfactory agreement between the official estimates of $NH_3$ emissions and measurements of the quantities of $NH_3$ and $NH_4$ present in air[16,17,18,19]. Emission estimates for $N_2O$ also yield very high uncertainty. Emission statistics indicate, however, a decrease by about 10% in the EU between 1998 and 1990[12].

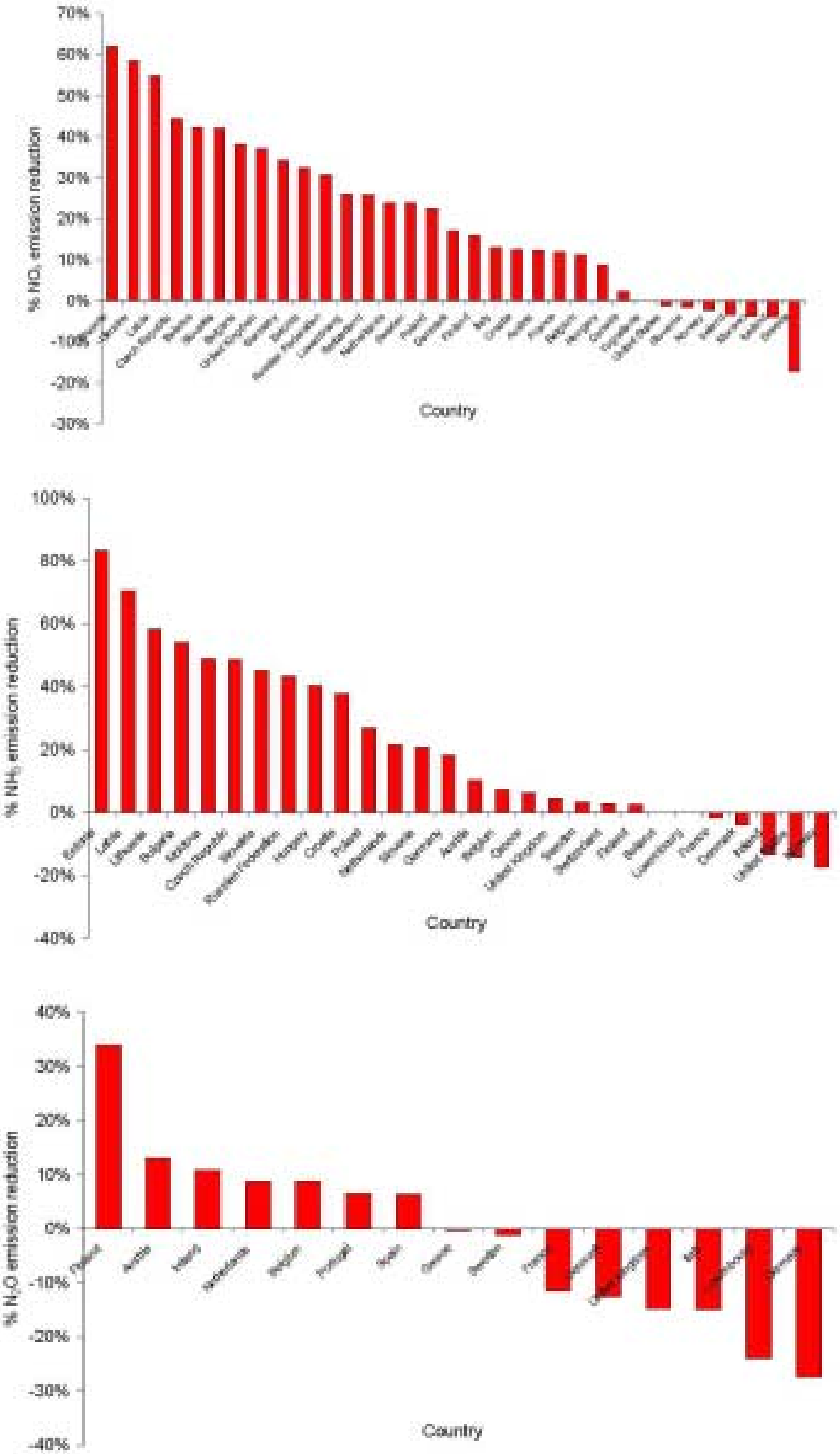

**FIGURE 3.** Changes in emissions (%) between 1990 and 1998 for $NO_x$ (A), $NH_3$ (B) and $N_2O$ (C). (Data from EMEP.)

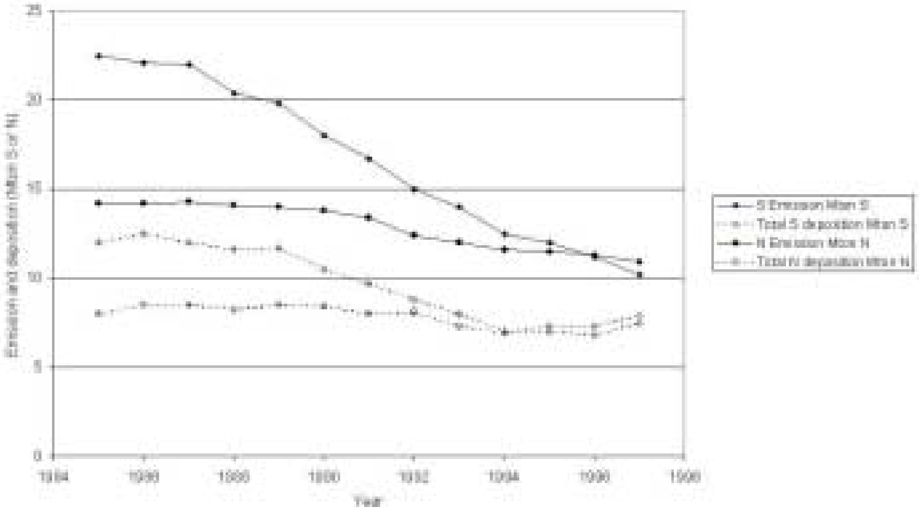

**FIGURE 4.** Total (modelled) deposition and estimated emission in Europe between 1985 and 1999 (Mton S or N per year).

There are notable regional differences in the emission trends during the last two decades. For sulphur most of the emission reductions between 1980 and 1990 appeared in NW Europe. After 1990 significant emission reductions have been achieved all over Europe. For $NO_x$ the picture is more complicated. Some countries report substantial emission reductions while a few report increases in their emissions. The largest emission reductions are reported from the former Eastern Europe where some countries report reductions of 40% and more (see Fig. 3). Most west European countries report emission reductions of 20–30% while in the Mediterranean Europe emissions were in 1998 about the same as in 1990. Emission reductions have been achieved in 1998 due to several measures. For $NO_x$ the main measure to reduce emissions is exhaust gas regulations introduced in the EU countries in about 1990, resulting in the application of three-way catalysts in gasoline cars. Even regulations on heavy-duty vehicles have caused emission reductions of $NO_x$. Furthermore, selective catalytic reduction technologies (SCR) with ammonia or urea as a reductor have been implemented in many combustion plants. In East Europe the main reason for the reductions is the closure of a large number of industrial plants. For ammonia and $N_2O$ the eastern European countries show the largest reductions, because of the decrease of agricultural activities.

## CONCENTRATIONS AND DEPOSITION

Every year EMEP updates deposition maps of for sulphur and nitrogen pollutants and ozone exposure maps based on meteorology driven theoretical models. The calculations are based on the gridded emission data in Vestreng and Støren[11]. Until recently, the model calculations were based on a Lagrangian model with a grid size of 150 km, with the emission inventory serving as input. Currently, the EMEP calculations are based on a new Eulerian model and EMEP is running the model for all previous years with appropriate emissions. The Eulerian model has some advantages over the Lagrangian model, such as the improvement of scale ($50 \times 50$ km$^2$) and a better description of vertical concentration profiles in the atmosphere. The Eulerian model calculations have so far only been done for 2 years and were not available for this paper. Fig. 4 shows the trend in calculated emission and deposition for total sulphur and nitrogen averaged over Europe according to the Lagrangian model. The deposition shows more year-to-year variation than for emissions due to the influence of meteorological factors, but the trend in deposition is less than that for emissions. During the years there is no trend in modeled N

deposition, whereas modeled emissions decreased by 21% ($NO_x$) and 14% ($NH_3$) in the same period. Possible reasons for this effect include nonlinearity in sulphur emission and deposition. Nonlinearity is the difference between the reduction in emissions of $SO_2$ and that in total S deposition, which is lower because of the change in atmospheric chemistry and surface affinities for sulphur. Sulphur emissions have decreased to a large extent in Europe and so is ammonium sulphate formation. This leaves more ammonia to form ammonium nitrates, which lead to higher nitrogen deposition.

There are some limited concentration data that are in accord with expectations, based on the emission trends, but are representative for a limited area only. Ambient concentration measurements from the EMEP network for total nitrate ($HNO_3$+$NO_3$) and total ammonium ($NH_3$+$NH_4$) are shown in Fig. 5. Also shown are $N_2O$ concentration measurements made in Ireland and at Samoa[12]. While the observations must be interpreted with care, the general picture from these observations is that concentrations decrease, but the extent is different in different locations in Europe. The measurements are not representative for all situations in Europe. In contrast, nitrous oxide ($N_2O$) concentrations show a steady increase. These are representative for a much larger area than Europe and reflect global emission trends.

Erisman and de Vries[20] made an overview of nitrogen deposition measurements in Europe and showed that there has been a small but significant decrease. For example, micro-meteorological observations at Speulder forest show a small downward trend in $NH_x$ deposition, reflecting the measures in the Netherlands taken to reduce emissions. Measurements of total oxidised ($NO_y$) and total reduced ($NH_x$) nitrogen fluxes in throughfall over Europe show a downward trend, if measurements between 1980–1993 and 1993–1997 were compared. Throughfall measurements for nitrogen, however, have to be interpreted with care because of the canopy exchange processes that take place[21].

Ozone ($O_3$) plays an important role in effects on humans (photochemical smog) and vegetation. Individual countries have their own ozone monitoring networks as well as taking part in international programs such as the EU-EUROAIRNET (to assess European air quality with respect to EU directives, Table 1) and EMEP. These data show that over Europe no clear trends are observed, however, there are indications that the maximum values are decreasing[6,22].

## PREDICTIONS FOR 2010

As deposition calculations are available for 150 × 150 km grid cells, a representative critical load value for an entire grid cell has to be determined. In order to do so the distribution of all critical load values for each ecosystem in the grid cell is made. From this distribution the 5th percentile is used as the critical load for that grid. In that way a map of critical loads is compiled (e.g., [23]). Since 1985, the area of critical load exceedance decreased by 76%[23]. The decrease is mostly due to the reduction of sulphur emissions and deposition, because nitrogen deposition was almost constant during the period. Consequently, there is no significant change in the total eutrophying exceedance for these areas. The critical load exceedance map used in the Gothenborg Protocol for 1990 is displayed in Fig. 6 for acidity and nitrogen and compared with the exceedance estimates predicted for 2010 taking account of the emission reductions agreed under the Protocol. The emissions of $NO_x$ and $NH_3$ are expected to be decreased by 50 and 12% respectively in 2010 relative to 1990[12]. This suggests that, through the Gothenborg Protocol, eventually the exceedance of critical acid loads will be reduced to a much smaller problem, with only a few grids where the exceedance is above acceptable levels. By contrast, for nitrogen the picture is very different: the areas with exceedance of critical nitrogen loads will hardly change.

It should be noted that the reduction in deposition below the critical loads does not always mean that the problem of acidification is over. In many areas, the acid deposition has caused serious and long-term damages to ecosystems that will take decades to recover[24]. Furthermore, it is important to add here that these maps may still underestimate the extent of critical load exceedance.

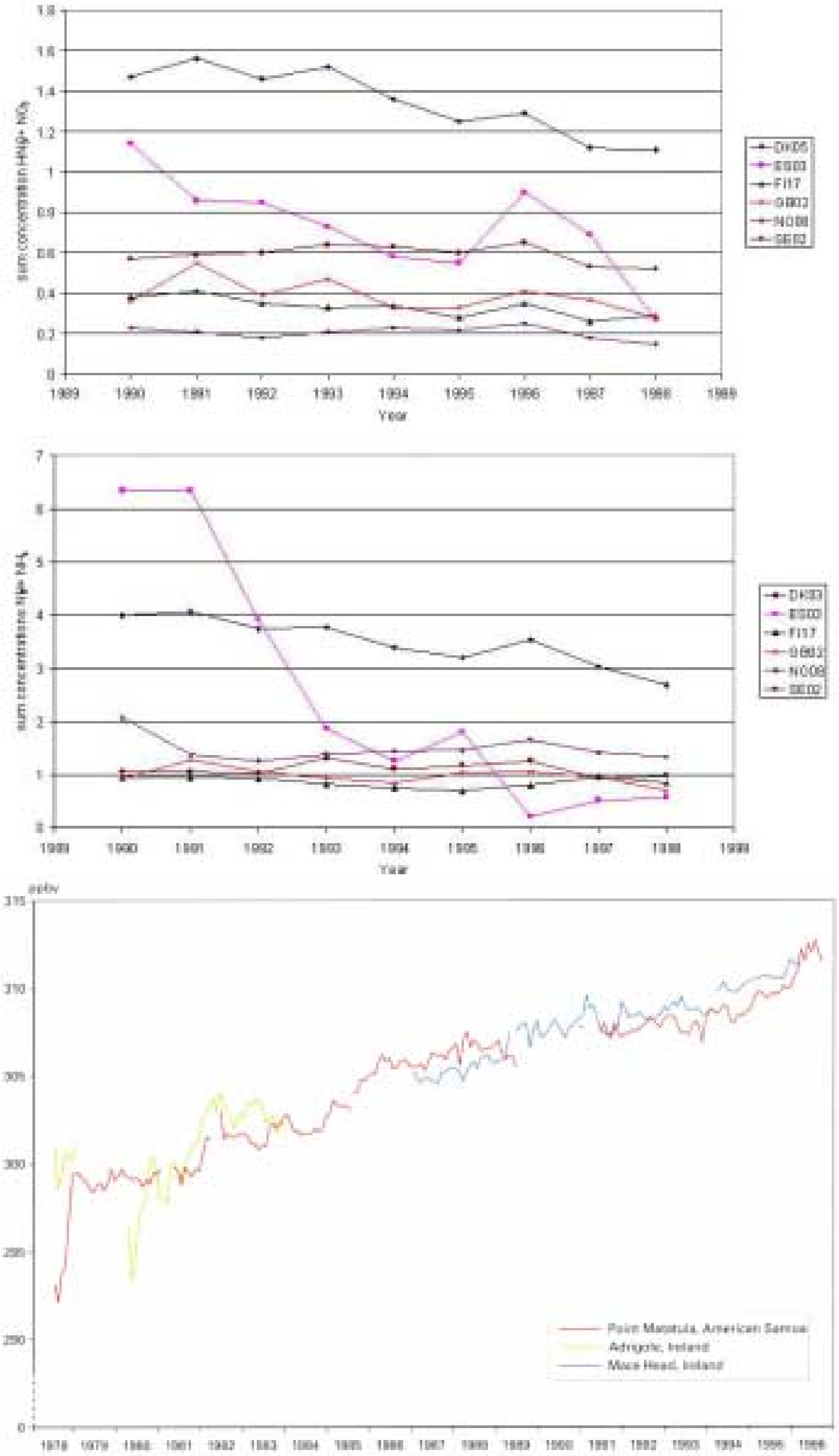

**FIGURE 5.** Ambient concentration measurements from the EMEP network for total nitrate ($HNO_3$+$NO_3$) (A) and total ammonium ($NH_3$+$NH_4$) (B) in µg m$^{-3}$ and $N_2O$ concentration (C) measurements in ppm made in Ireland.

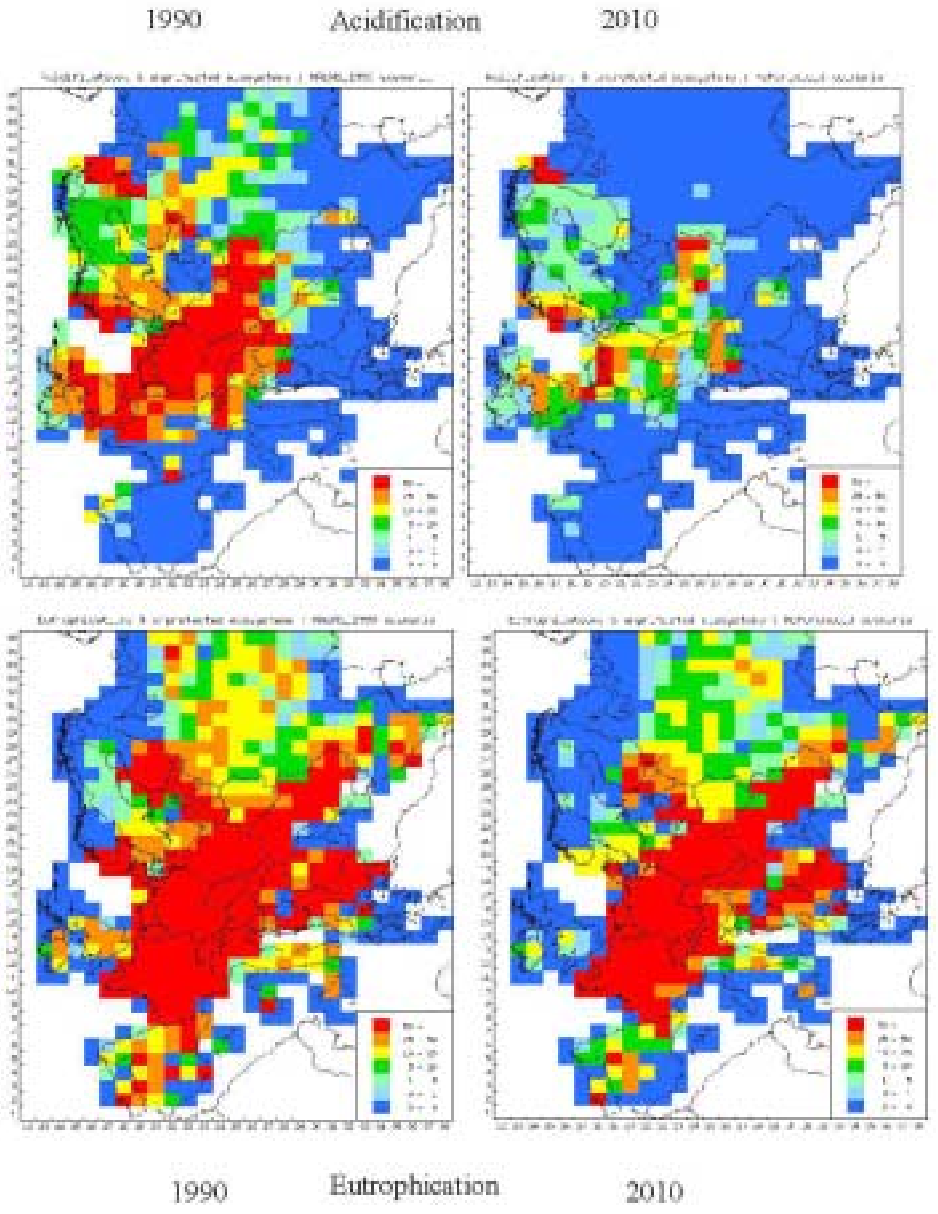

**FIGURE 6.** Exceedances of critical loads for acidity and nitrogen in 1990 and for the Ref-scenario developed to underpin the Gothenburg protocol. (data from IIASA, Austria).

This arises because of a correlation of ecosystem specific deposition rates and the sensitivity of ecosystems, which is currently not properly treated in the European scale modelling of EMEP. For example, deposition velocities ($V_d$) of nitric acid ($HNO_3$) are larger to forest vegetation than to short canopies, while $V_d$ values for $NH_3$ are larger for N sensitive moorland vegetation compared with agricultural land where the compensation point lowers the deposition rate and can even cause emissions[25,26].

It is expected that global background ozone concentrations will continue to rise due to increased precursor emissions, particularly in Eastern Europe, Asia and countries of the Southern hemisphere, but that emission control strategies (see Table 1) in N America and Europe will lead to a reduction in peak episodic concentrations in those regions. The implications for ozone critical level exceedance across Europe in 2010 have been studied by EEA and EMEP. In general the population exposed to potentially harmful levels of ozone is expected to decline, particularly on the north and west fringes of Europe, but most of the regions vegetation will still be exposed to considerable exceedances of critical levels[22]. The scenarios for 2010 are however very sensitive to the development of the background concentrations.

Scenarios for $N_2O$ show that the European emissions will grow by about 10% in 2010 relative to 1990 and concentrations of $N_2O$ in air will increase accordingly. Given its increasing relative contribution to transboundary air pollution and radiatively active gas concentrations in air, N will be the most important component for the coming years. Other areas, which will be important, are ozone levels, particulate matter and effects on human health (not addressed here).

## NEED FOR AN INTEGRATED POLICY TO DECREASE NITROGEN IN THE ENVIRONMENT

Each of the emitted gases participate in the cascade of processes leading to a series of inter-related effects, for example with deposition of reactive nitrogen ($NO_y$, $NH_x$) or fertiliser N inputs to ecosystems impacting on production, biodiversity, carbon sequestration, water quality and $N_2O$ emissions. Nitrogen in its various chemical forms plays a major role in a great number of environmental issues. It contributes to acidification and eutrophication of soil, groundwater and surface waters, decreasing ecosystem vitality and biodiversity and causing groundwater pollution through nitrate and aluminum leaching. Nitrogen compounds play an important role in carbon sequestration, global change, formation of ozone, oxidants and aerosols, potentially posing a threat to human health and affecting visibility. The cascade of effects starts at the local scale (human or ecosystem exposure to high gas, or particle concentrations in the atmosphere or nitrate concentrations in soils and groundwater). Through long-range atmospheric transport, river transport or groundwater transport, the effects extend from regional to continental (acidification, eutrophication, carbon sequestration, aerosols) and even global dimensions ($N_2O$)[27]. The cascade of effects depends on the N status of a region: This may be defined as the amount of excess N in the system (or region) causing effects at different levels in the cascade of effects[28,29]. If the N excess increases, the risk on the number of effects in the cascade increases (the cascade length increases). At the same time the area that is affected by N pollution increases (higher contribution to long-range transport or $N_2O$ emissions).

While the linkages in the cascade effect still require to be quantified at the different scales, the available information already highlights its importance. In the Netherlands it was concluded from a study where monitoring data were compared with model estimates based on emission calculations, that some measures to reduce the emissions were not as effective as had been predicted[16,29]. It is hypothesised that the main reason for this is the high N status in the Netherlands (average fertilisation rates of more than 400 kg/ha/year)[17,29]. The measures to decrease $NH_3$ emissions are primarily aimed at keeping N in the manure until the manure is incorporated into the soil by injection or ploughing, so that the plants can use the reactive N. In a situation with an overload, however, the N is not used efficiently and a large fraction is released in some reactive form back to the atmosphere or to the groundwater. This is part of the cascade effect. The next stage of the cascade is reached when the ‘optimum’ of the accumulated N is exceeded and the reactive N flows into another compartment of the ecosystem where it is accumulated, etc. Only at the beginning of the cascade is the form of reactive N of importance. In the next stages, it will be transformed either in the oxidised or reduced form and the origin is of little importance, whether it comes through the atmosphere or directly from manure or through mineralisation or nitrification in the soil.

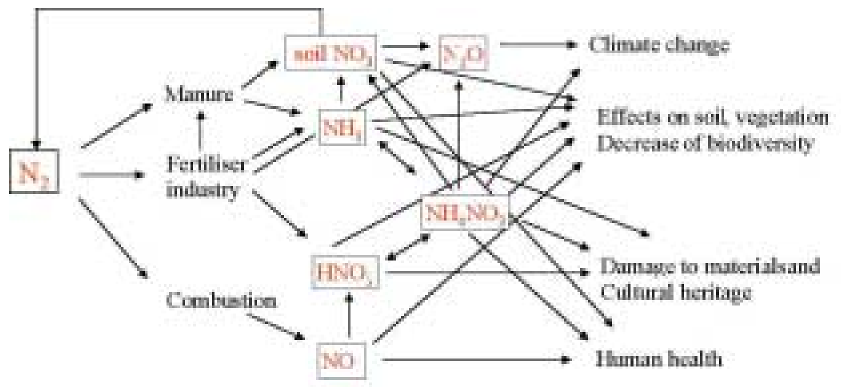

**FIGURE 7.** Different sources and effects of nitrogen in the environment and their relations.

The cascade effects will continue until N is either:

- diluted into harmless concentrations,
- immobilised in the ecosystems in a way that it only will be available over a very long term, or
- denitrified to molecular nitrogen.

The consequence of this is that an integral approach for N is necessary.

The main policy relevant items are displayed in Fig. 7, together with the three main sources of N compounds in the environment: 1) production of manure, through input or cycling of N rich feed in agriculture; 2) fuel combustion, and 3) fertiliser production. The key themes related to these issues are listed below, noting in brackets the relevant agreements, with the instruments of prime importance noted by * (after Sutton et al.[10]):

1. *Climatic change*: a) emissions of $N_2O$, which cause global warming and b) aerosols (secondary formed), which have a cooling effect (1*, 2*, 3*, 4, 5, 6, 7).
2. *Effects of elevated $CO_2$:* direct effects of enhanced $CO_2$ include altered plant productivity (with the effects modified by atmospheric N deposition) and, potentially, species competition change in semi-natural ecosystems (1*, 2*, 3*, 4, 5, 6, 7).
3. *Acidification:* long-range transport of primary gases $SO_2$, $NO_x$, $NH_3$ and derivatives (aerosols, acid gases) leading to acidification of soils and freshwaters (4, 5, 6, 7, 8*, 9*, 10*, 11*, 12*, 13*).
4. *Eutrophication*: long-range transport of reduced nitrogen ($NH_x$) and oxidised nitrogen ($NO_y$) compounds (including $NH_3$, $NH_4^+$, $NO_x$, $HNO_3$, $NO_3^-$, $HNO_2$, etc.), leading to soil and plant community changes particularly in semi-natural ecosystems (5, 6, 7, 8*, 9*, 10*, 11*, 12*, 13*).
5. *Regional ozone* and *particle formation*: through emissions of primary gases ($SO_2$, $NO_x$, $NH_3$, VOC). Peak concentrations of ‘episodes’ affecting human health, ecosystems, crops and forest trees (5, 7, 9*, 10*, 12*, 13*).
6. *Tropospheric chemistry changes*: through emissions of primary gases ($NO_x$, VOC, $SO_2$, $NH_3$) and formation of $O_3$, other photochemical products and aerosols, affecting ‘background’ levels to which the episodes add (5, 7, 9*, 10*, 12*, 13*).

[1] UN Framework Convention on Climate Change – Kyoto Protocol
[2] UN Montreal Protocol
[3] EU Methane Strategy
[4] Helsinki Agreement on Forest Protection
[5] UN Biodiversity Convention
[6] EU Agenda 2000
[7] EU Habitats Directive (Natura 2000)
[8] EU Directive on Integrated Pollution Prevention and Control
[9] EU Acidification Strategy
[10] UNECE Convention on Long-Range Transboundary Air Pollution (CLRTAP)
[11] CLRTAP: 2nd Sulphur Protocol
[12] CLRTAP: multi-pollutant, multi-effect ‘Gothenburg Protocol’

Dealing with the whole N cycle is clearly a major challenge. As a result, most research has addressed the different N forms and pollution issues separately. The same holds true when it comes to developing abatement policies. These are currently widely separated, with for example, the EU Nitrates Directive dealing with eutrophication of ground and fresh waters from agriculture, the UNECE Convention on Long-Range Transboundary Air Pollution (CLRTAP) and the planned EU National Emissions Ceilings (NEC) Directive dealing with regional air pollution (acidification, eutrophication and photochemical oxidants), and the Framework Convention on Climate Change (FCCC) dealing amongst other with emissions of $N_2O$ as a greenhouse gas[10,30].

As shown, the relative contribution of N compounds to the total deposition of pollutants is becoming more important. Furthermore, it was shown that acidification will be substantially reduced after full implementation of the Gothenburg protocol in 2010. On the contrary, eutrophication will not diminish greatly. Nitrogen is difficult to abate, because of the intimate linkages between its various forms: this applies equally to agriculture, where reducing $NH_3$ emissions may increase $N_2O$ and nitrate, as to transport emissions, where reducing $NO_x$ emissions is leading to increased $NH_3$ and $N_2O$ emissions. Although some technical measures can be implemented relatively cheaply, many are expensive. The most difficult sources to abate are vehicles, small combustion sources and agricultural sources. Large industrial point sources are much easier to abate, because technology is well developed, even though it is rather expensive. Of importance is also the expected growth in food production and energy use. The global population is expected to grow continuously while the agricultural area used for food production cannot increase indefinitely. Therefore, the use of fertilisers is expected to grow exponentially. Furthermore, the standards of living are expected to increase in developing countries, also leading to the demand for more intensive agriculture regions. The prospects for emission reduction are therefore limited. A further problem, which is of particular relevance for reduced nitrogen, is that the emissions and deposition budgets even at a country scale are highly uncertain. In one of the most intensively studied countries, the Netherlands, there is still an ‘ammonia gap’ reflecting a difference between the estimated emissions based on activities and emission factors, and emission rates derived from measurements[16,29].

While some progress toward integration has been made, notably within the CLRTAP, the linked nature of the N cycle demonstrates the limitations of such separation, both in the research and policy development. The result is that important issues of synergy and conflict between mitigation strategies are frequently missed[10]. Nitrogen components form a central role in this system with many interrelations, as shown in Fig. 8. Grennfelt et al.[31] first illustrated the links between sources of regional air pollutants, pollution issues and the impacted receptors. Fig. 8, which is modified from Sutton et al.[10], extends this to consider the interactions with radiatively active traces gases and toxic pollutants. The interrelations between sources, issues and effects where N plays a role are given in grey in Fig. 8. This figure shows that it is likely more effective to couple and adjust abatement strategies resulting in a reduction of more than one effect.

Because N is a central element, a first step into integration is the development of a N management strategy to prevent systems from negative (cascade) effects. The cascade effect of N has so far not been addressed either in research or in environmental policy. To date negotiations developing mitigation strategies have been focused on single compounds or issues with a lack of interrelations or linkages or as in the case of the recent approaches for transboundary air pollution, only on compounds that are emitted to the atmosphere and cause effects on a regional scale.

The issues to be included in a joint N management strategy include negative effects such as acidification, eutrophication, greenhouse gas emissions, particle exposure and groundwater pollution, but also the positive effects in the form of increased food and wood production and increased carbon sequestration in forests. One of the consequences of the cascade effect is that by limiting the effects of one issue, e.g., injection of manure to decrease ammonia emissions, the N in

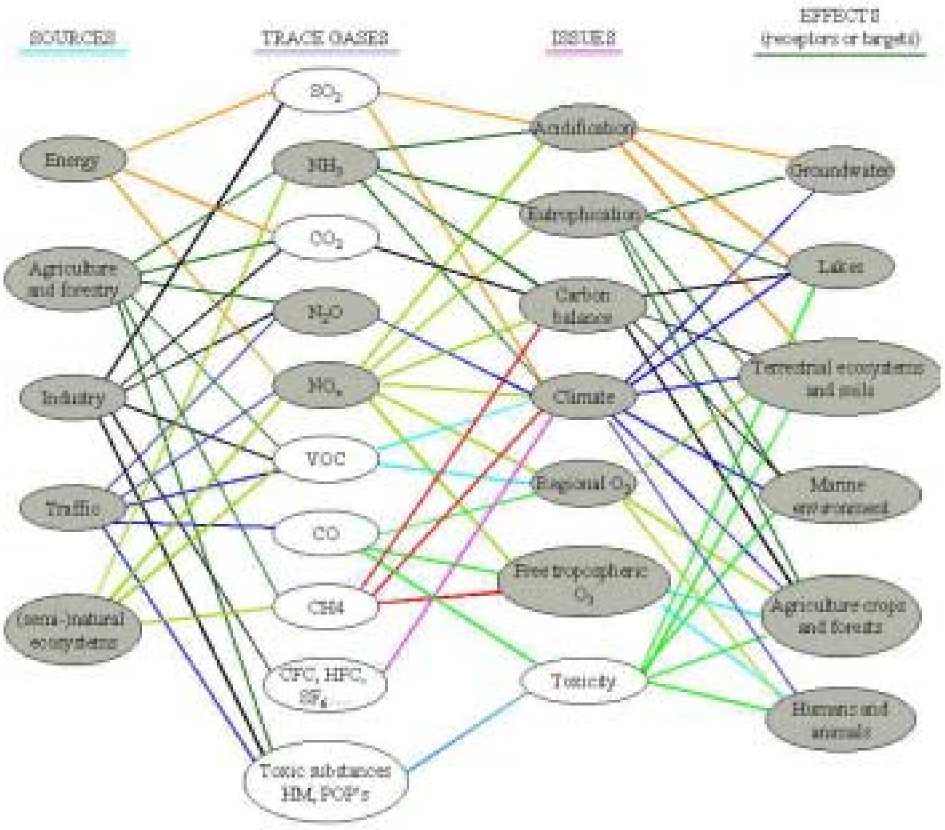

**FIGURE 8.** The interrelations between sources, compounds, environmental issues and receptors, with a focus on the atmospheric components. Ellipses shown in grey indicated problems linked to the N cycle. Modified from Refs.[31,10].

reactive form can contribute to other issues instead, such as in this case the increase of nitrate leaching to groundwater or $N_2O$ emissions. An integrated N policy, which reduces the cascade effects and therewith contributes to the improvement of several effects in one time, would therefore be highly beneficial to society as a more effective form of pollution control. Furthermore, such an integrated policy might be much more cost effective and feasible than current approaches. With hindsight this might appear obvious. Clearly, there is a requirement to provide a system-based analysis that addresses the linkages inherent between these different N pollutants and their control. Currently, such an integrated approach is conspicuous by its absence, and it is a major challenge for both scientists and policy makers to address the level of integration necessary.

A further reason why an integrated approach to N has not been taken is institutional limitation at national, continental and global levels. Policy structures have developed to deal with specific problem issues, e.g., transboundary pollution (UNECE CLRTAP), global climate change (UN FCCC), marine pollution (Oslo and Paris Commissions) or nitrate in groundwater (EU Nitrates policy). The clear focus of each has been helpful to ensure good progress in the different areas. At national levels, and increasingly in the EU, the policy needs also drive the research agenda. The result is that the research becomes tuned to a set of clearly *separated* policy issues. As the often-conflicting interests between these policy structures become increasingly apparent, there is a need for an increased level of institutional cooperation.

An integrated approach is necessary to reach efficient and effective solutions for environmental problems related to the increased production and accumulation of reactive N in the environment. Based upon current knowledge and experience with integrated assessment modelling for the development of the Gothenburg protocol, such an approach is feasible. The integrated approach should lead to improved protection against excess reactive N within acceptable conditions for society and within acceptable costs. An example of such a policy could be the development of reactive N production ceilings for regions in Europe based on critical limits for the different effects associated with N pollution. Together with measures to reach these ceilings, this would provide a solid basis to reach the goal[29]. Such an Optimum Nitrogen Management Strategy for Europe could be applied in the EU and on a national level to reach several environmental targets. The production ceilings might be also used as conditions for trades in production permits. As N has two sides: economical value in N-poor regions and a waste product in N-rich areas, such a trade might be economically feasible. One of the problems at this moment is that N (fertiliser) is inexpensive. The setting of production ceilings needs to be based on the cascade of effects in different compartments and to the different *target groups*. This method would provide a direct link between the environmental constraints and the maximal production of reactive N. It could be used to optimise the distribution of production over Europe (or regions) given the current infrastructure, etc. The direct link also demonstrates the limits to production and the related agricultural and industrial activities given the technological opportunities. Thus such a management strategy also provides a tool for the ‘negotiation’ between the environment on the one hand and the human activities at the other: if the environmental limits are set, then the activities are bound to a maximum. Under a permitting system this maximum would only be increased when permits from other regions are bought or when the environmental limits are relaxed. The feasibility of such a strategy or related alternatives needs to be grounded in the perspective of the N cycle on a European scale, drawing both policy and sector users into the debate. While there is inherent exchangeability at a regional and global scale allowing such a trading system to apply, it must be noted that some constraints are necessary where significant impacts of nitrogen occur at a local level at the start of the nitrogen cascade.

## CONCLUSIONS AND FUTURE NEEDS

From the analysis presented in this paper it can be concluded that although some progress is being made in decreasing N emissions, the future perspectives for reaching environmental targets are not very promising. For $NH_3$ the Gothenburg protocol is only the first small step to limit emissions, and substantial critical load exceedance is still expected for 2010. The current uncertainty in $NH_3$ emissions and in the national balances, together with the doubts about the effectiveness of different measures makes reducing the uncertainties a priority in order to reach political agreement on further $NH_3$ emission reductions in the coming years. For $NO_x$ more reliable and cost-effective technologies are becoming available to reduce emissions. However, there is a trade-off with extra energy use or other emissions to the atmosphere (e.g., $NH_3$). For $NO_x$ it would be better to focus on measures to decrease $CO_2$ emissions, because in many options $NO_x$ will be decreased at the same time. For the transport sector the growth in traffic has partly compensated the effect of exhaust gas regulations, while exhaust $NH_3$ and $N_2O$ emissions have increased as by-products of the catalytic removal of $NO_x$. The further abatement of transport N emissions may be difficult. An attractive option for combating these N emissions would be the change of a gasoline society into a hydrogen society. If hydrogen could be used as a fuel for fuel cells in cars and local energy production, the N emissions could be concentrated in large facilities where $H_2$ is produced, allowing for more efficient pollution control. Furthermore, if in such a situation, in the long-term, $H_2$ could be produced from renewable sources, large reductions could be achieved. For $N_2O$, emissions are predicted to increase in the coming years. For emissions of $N_2O$ due to nitric acid production, there are technologies available that can be implemented as soon as the economical incentive is there. For agriculture, emissions will be difficult to decrease by more than a small amount using only technical measures that affect the fate of N.

Important factors associated with increased N emissions are the need for more food, the change in food patterns in developing countries (more meat, more luxurious food) and the need for more energy. While the population is growing fast, and accordingly the need for food is increasing, the total land area available for growing this food is only minor increasing. More fertilisers are therefore needed to grow the food. Furthermore, human need for energy will increase substantially. These are important factors that will determine the future ‘waste’ of reactive N into the environment.

In this paper an integrated N approach is proposed with N production limits as an instrument to prevent critical environmental limits being exceeded. Such an approach would show where the main emphasis should be placed and what measures should be taken. These are related to limiting the production of reactive N, the import and/or use in a specific region. The scientific understanding of behaviour of N in the environment and its emissions and transformations, needs to be improved in order to make a quantitative analysis necessary to underpin the estimation of reactive N-ceilings. The main emphasis in future research should be on:

- quantifying the cascade effect and the optimum N levels for different systems
- knowledge on key-processes and the dynamics in N-flows that are still very uncertain: denitrification, soil emissions, ammonia emissions, the role of $NH_3$ in atmospheric chemistry (nonlinearities, re-emissions, etc.) and run-off to surface water and marine systems
- improvement of understanding of emissions of $NH_3$ and $N_2O$, especially in agriculture in relation to different management options
- improvement of understanding of biosphere – atmosphere interactions and the dynamics
- deriving optimum levels or critical limits related to different environmental N-effects
- development of source — receptor and receptor — source relations together with optimisation techniques (based on economics) and decision support systems to derive maximum N ceilings

Furthermore, the integrated nitrogen approach could be extended by including the links to the carbon, sulphur, and phosphorus cycles in order to make it an integrated environmental approach.

## ACKNOWLEDGEMENTS

We gratefully acknowledge funding from the EU under the GRAMINAE project (ENV4-CT98-0722) (JWE, MAS), from the UK Natural Environment Research Council programme on Global Nitrogen Enrichment (GANE) (MAS), and the UK Department of Environment, Food and Rural Affairs (MAS).
